# Single Nucleotide Polymorphism Analysis Indicates Genetic Distinction and Reduced Diversity of Swine-Associated Methicillin Resistant *Staphylococcus aureus* (MRSA) ST5 Isolates Compared to Clinical MRSA ST5 Isolates

**DOI:** 10.3389/fmicb.2018.02078

**Published:** 2018-09-11

**Authors:** Samantha J. Hau, Anna Allué-Guardia, Brigida Rusconi, Jisun S. Haan, Peter R. Davies, Timothy S. Frana, Mark Eppinger, Tracy L. Nicholson

**Affiliations:** ^1^Department of Veterinary Diagnostic and Production Animal Medicine, College of Veterinary Medicine, Iowa State University, Ames, IA, United States; ^2^South Texas Center for Emerging Infectious Diseases, University of Texas at San Antonio, San Antonio, TX, United States; ^3^Department of Biology, University of Texas at San Antonio, San Antonio, TX, United States; ^4^Department of Veterinary Population Medicine, College of Veterinary Medicine, University of Minnesota, Saint Paul, MN, United States; ^5^National Animal Disease Center, Agricultural Research Service, United States Department of Agriculture, Ames, IA, United States

**Keywords:** LA-MRSA, *Staphylococcus aureus*, whole genome sequence (WGS), single nucleotide polymorphism (SNP) typing, phylogenetic analysis, swine, mobile genetic elements, agriculture

## Abstract

Livestock associated methicillin resistant *S. aureus* (LA-MRSA) are lineages adapted to livestock species. LA-MRSA can be transmitted to humans and public health concerns exist because livestock may be the largest MRSA reservoir outside of hospital settings. Although the predominant European (ST398) and Asian (ST9) lineages of LA-MRSA are considered livestock adapted, North American swine also harbor ST5, a globally disseminated and highly pathogenic lineage. This study applied whole genome sequencing and single nucleotide polymorphism (SNP) typing to compare the population structure and genetic relatedness between swine associated and human clinical MRSA ST5 isolates. The established high-resolution phylogenomic framework revealed that LA-MRSA and human clinical MRSA ST5 are genetically distinct. LA-MRSA isolates were found to be clonal within farms, while greater genome diversity was observed among sampled clinical MRSA ST5. Analysis of the accessory genome demonstrated that LA-MRSA ST5 isolates and clinical MRSA ST5 isolates harbor different AMR genes and virulence factors, consistent with the SNP analysis. Collectively, our data indicate LA-MRSA and clinical MRSA ST5 isolates are distinct and the swine reservoir is likely of minimal significance as a source of clinical MRSA ST5 infections.

## Introduction

*Staphylococcus aureus* is a commensal organism found in the nasopharynx and on the skin of humans and other mammals. It can also cause infections in these hosts and cause a range of diseases from mild skin and soft tissue infections to severe systemic infections. The treatment of *S. aureus* infections is hampered by the development of antimicrobial resistance (AMR) in these isolates, such as the acquisition of the SCC*mec* element, which confers methicillin resistance. These isolates are designated as methicillin resistant *S. aureus* (MRSA) and have become a significant burden for the health care system (Klevens et al., 2007; DeLeo and Chambers, 2009).

Methicillin resistant *S. aureus* isolates are classified based on epidemiologic characteristics into hospital acquired MRSA (HA-MRSA), community acquired MRSA (CA-MRSA), and livestock associated MRSA (LA-MRSA) (Stefani et al., 2012). These subsets are defined by the source of the isolate and the isolates within each subset tend to share genotypic and phenotypic characteristics, such as degree of AMR and virulence factors. Specific lineages or sequence types (STs) tend to predominate within each group, although there are regional variations. For example, in the United States, ST5 and ST8 are major HA-MRSA and CA-MRSA clones, respectively (Bal et al., 2016).

Livestock associated MRSA became a significant public health concern in 2005, when the first report linked MRSA ST398 to swine production facilities (Voss et al., 2005). Further investigations indicated swine may serve as the largest reservoir for MRSA outside of hospital settings and motivated considerable research into the potential health risks associated with LA-MRSA (Schijffelen et al., 2010). Subsequent research showed the most prevalent lineage of LA-MRSA varied based on geographic location. In European swine populations, ST398 is the most common lineage (Andreoletti et al., 2009), while the ST9 lineage predominates in swine in most Asian countries (Wagenaar et al., 2009). In the United States, the swine population was found to harbor more diverse STs with isolates of the ST398, ST9, and ST5 lineages (Molla et al., 2012; Frana et al., 2013; Sun et al., 2015). The presence of MRSA ST5 isolates in United States swine herds raised additional public health concerns because the ST5 lineage, unlike the ST398 and ST9 lineages, is not considered livestock adapted but is a highly successful and globally disseminated MRSA lineage in and out of hospital settings (Monecke et al., 2011; Fitzgerald, 2012; Pantosti, 2012). The widespread success of the ST5 lineage has been attributed to its capacity to acquire mobile genetic elements (MGEs) that harbor virulence factors or AMR genes (Monecke et al., 2011).

Phylogenetic studies employ whole genome sequencing (WGS) technology and data analysis to better understand the epidemiology, origin, and evolution of bacteria (Price et al., 2012; Uhlemann et al., 2012; Eppinger et al., 2014; Sahibzada et al., 2017). In the case of LA-MRSA, these techniques have been used to assess the relatedness of isolates from the ST398 lineage. LA-MRSA ST398 isolates clustered separately from human ST398 isolates and are suspected to have evolved from an ancestral methicillin susceptible ST398 clade in humans (Price et al., 2012). Similar investigations into the CC97 lineage indicated that isolates causing clinical disease in humans comprise a sub-clade of the LA-MRSA isolates that may have developed an increased capacity for infecting and causing disease in humans (Spoor et al., 2013). WGS analysis can be used to assess isolate relatedness or determine genetic characteristics that define subsets of isolates, including information about the MGEs harboring virulence factors or AMR genes. Through single nucleotide polymorphism (SNP) discovery within the core genome, the accuracy and resolution power is available to determine phylogenetic relationships and distinguish isolates within highly homogenous lineages, which provides insight in epidemiological investigations (Eppinger et al., 2011, 2014; Rusconi et al., 2016; Sahibzada et al., 2017).

Although previous reports have examined the genetic diversity and relatedness of MRSA isolates from livestock species and humans within the ST398 and CC97 lineages (Price et al., 2012; Spoor et al., 2013), to date there are no reports using WGS data to evaluate the genetic diversity of MRSA ST5 isolates obtained from swine and human sources. In this study, we used SNP analysis of the core genome to evaluate the population structure and genetic diversity of LA-MRSA ST5 isolates from a variety of swine associated sources and clinical MRSA ST5 isolates from humans with no swine contact to investigate the potential for LA-MRSA ST5 isolates to act as a source for human infections or as a source for MGEs harboring virulence factors or AMR.

## Materials and Methods

### Isolate Acquisition

Swine associated LA-MRSA ST5 isolates were obtained from samples collected while visiting pork farms in the Midwest United States as part of field case investigations completed by Iowa State University (ISU) (Frana et al., 2013). Sources for these isolates were swine nasal swabs (38 isolates), the environment within swine facilities (25 isolates), and humans with short-term contact with swine (9 isolates) (Frana et al., 2013). Isolates from healthy practicing swine veterinarians were provided by the University of Minnesota (UMN). The isolates (*n* = 9) were acquired from swine veterinarians who participated in a longitudinal study focusing on *S. aureus*. Veterinarians resided in areas of intensive swine production in the Midwest and Southeast United States and had regular (i.e., greater than twice per week) professional contact with pigs (Sun et al., 2017). MRSA ST5 isolates from humans with no swine contact were obtained from medical centers associated with the University of California Irvine (UCI) (*n* = 64) (Hudson et al., 2013) and the University of California, San Francisco (UCSF) (*n* = 8). Isolates were characterized for MLST and *Spa* typed prior to acquisition (Frana et al., 2013; Hudson et al., 2013; Sun et al., 2017). Specific isolate information can be found in **Supplementary Table S1**. All isolates were either obtained from samples collected as part of previous studies and obtained through written consistent (Frana et al., 2013; Hudson et al., 2013; Sun et al., 2017) or were obtained from samples submitted as part of field case investigations and did not require Institutional Animal Care and Use Committee (IACUC) approval.

### Whole Genome Sequencing

Draft genomes were generated as previously described (Hau et al., 2017a,b,c,d,e,f). Briefly, total genomic DNA was extracted from isolates grown in Trypticase Soy Broth (BD Biosciences, Sparks, MD, United States) using a High Pure PCR Template Preparation Kit (Roche Applied Science, Indianapolis, IN, United States). The Nextera XT DNA sample preparation and index kit (Illumina, San Diego, CA, United States) was used to generate paired-end DNA libraries with 250-bp read length that were sequenced using the MiSeq v2 500 Cycle reagent kit on the Illumina MiSeq platform (Illumina, San Diego, CA, United States). Sequence reads were assembled with MIRA v. 4.0.2^1^ and annotated using the NCBI Prokaryotic Genome Annotation Pipeline^2^. Draft genomes and sequence reads have been submitted to GenBank and the Sequence Read Archive (SRA). Accession numbers are provided in **Supplementary Table S1**.

### Core Genome SNP Discovery

For reference based SNP discovery we used a custom developed pipeline implemented on Galaxy (Goecks et al., 2010), that was successfully applied for the high resolution genomic epidemiology profiling of various microbial human pathogens (Eppinger et al., 2011, 2014; Rusconi et al., 2016). Strategies and phylogenetic principles have been described in detail in Rusconi et al., 2016. The MRSA core genome in this study is defined in the samples as the set of genic and intragenic regions that are present in all genomes, not repeated, and do not contain MGEs (which evolve at different rates and are not indicative of evolutionary relationships), such as phages, IS elements, genomic islands or plasmids. These excluded regions were determined for the reference genome Mu50 as follows: NUCmer was used to detect repeat regions by running the reference against itself (Delcher et al., 2003), integrated bacteriophages were identified using PHAST (Zhou et al., 2011), ISFinder was used for detection of insertion sequences (Siguier et al., 2006), and antibiotic resistance cassettes were detected with ResFinder (Zankari et al., 2012). The SNP discovery and verification pipelines are implemented on Galaxy, and contain the following modules: (i) SNP discovery and typing, (ii) SNP curation, (iii) SNP annotation, (iv) SNP distribution, and (v) SNP phylogeny.

#### SNP Discovery and Typing

Illumina reads of the 72 MRSA ST5 isolates from humans with no swine contact and 82 LA-MRSA ST5 were uploaded into Galaxy along with two ST5 representative closed genomes: a HA-MRSA isolate from Japan (Mu3) and a representative poultry-adapted MRSA ST5 isolate (ED98) (Hiramatsu et al., 1997; Lowder et al., 2009). For read-based SNP discovery, reads were aligned with Bowtie2 to the reference genome Mu50 (Langmead and Salzberg, 2012). The resulting alignments were processed with Freebayes using the following threshold settings: mapping quality 30, base quality 20, coverage 30, and allelic frequency 0.9 (Garrison and Marth, 2012). The two closed representative genomes, Mu3 and ED98, were analyzed using the contig-based workflow. Briefly, a panel of SNPs for each genome was obtained by aligning the genome against the reference strain, Mu50, using NUCmer. SNPs were called with delta-filter and show-snps distributed with the MUMmer package (Delcher et al., 2003).

#### SNP Curation

Several SNP curation strategies were used to correct for false positive calls (Eppinger et al., 2011, 2014). First, reads were mapped against the reference genome Mu50 and false positives identified by Freebayes with the settings described. If reads were unavailable (Mu3 and ED98), the post-assembly workflow generated a reference-based NUCmer alignment and extracted SNPs as described above with filtering of false SNPs. SNPs located within excluded regions (repeat regions, bacteriophages, resistance cassettes, and IS elements) were removed. SNPs were further curated by extracting the 40 nucleotides surrounding each predicted SNP in the reference genome and completing a nucleotide BLAST against the query genomes (Altschul et al., 1990). Finally, resulting alignments were parsed to remove SNP locations derived from ambiguous hits (≥2), low alignment quality or misalignments, non-uniformly distributed regions, and InDels, as previously described (Myers et al., 2009; Eppinger et al., 2011, 2014). Also, multinucleotide insertions and deletions of polymorphic bases were not considered SNPs and were excluded.

#### SNP Annotation

The curated cataloged SNPs from each query genome were merged into a single SNP panel that reported the allele, genic/intergenic status, SNP position, and annotation (Uhlemann et al., 2012; Spoor et al., 2013; Rusconi et al., 2016). This SNP discovery and validation pipeline allows for rapid typing of strains of unknown provenance by interrogating the captured allelic states from established SNP panels (Eppinger et al., 2011).

#### SNP Distribution

From the distribution of SNPs along the Mu50 chromosome, potential mutational hotspots and genes under positive selection could be identified using custom scripts implemented on Galaxy (Giardine et al., 2005; Myers et al., 2009; Goecks et al., 2010).

#### SNP Phylogeny

The identified curated SNP panel was used for phylogenetic reconstruction by maximum parsimony with PAUP v4.0a146 with 100 bootstrap replicates (Wilgenbusch and Swofford, 2003). The SNP tree was visualized in Geneious (vR9) and the majority consensus tree was built in Mesquite (Kearse et al., 2012; Maddison and Maddison, 2017). Tree decorations were added using Evolview (He et al., 2016). Calculation of the consistency index for each SNP allowed for identification of parsimony informative SNPs and flag homoplastic SNPs, as described in our previous works (Eppinger et al., 2014; Rusconi et al., 2016). A Maximum Likelihood phylogenetic tree was additionally inferred using RAxML v8.2.11 with the GTR CAT model and 100 bootstrap replicates (Stamatakis, 2014). The consensus tree was built and visualized in Geneious (vR9) (Kearse et al., 2012).

### Mobile Genetic Element Analysis

Mobile genetic elements harboring AMR genes and virulence factors were detected as described previously and verified using Geneious 9.0.5 (Hau et al., 2015, 2017g; Hau, 2017). AMR genes detected with ResFinder were verified through draft genome analysis (Hau, 2017). The SCC*mec* type was determined using PCR and confirmed *in silico* (Hau et al., 2017g). Immune evasion genes associated with the β-hemolysin converting bacteriophage were identified in these isolates using PCR and confirmed *in silico* (Hau et al., 2015).

## Results

### Isolate Provenance and Sequence Information

Swine associated LA-MRSA ST5 isolates were obtained from nasal swabs of healthy pigs or humans, none of which exhibited signs of MRSA infection, or from swabs of the environment within swine facilities. Clinical MRSA ST5 isolates were obtained from patients with MRSA related disease at two urban, university affiliated hospitals in California where the likelihood that patients had contact with swine was considered negligible. Draft genomes confirmed the MLST data indicating all isolates were ST5.

Draft genome sequences were generated for the 81 LA-MRSA ST5 isolates and 72 MRSA ST5 isolates from humans with no swine contact (**Supplementary Table S1**). Genome statistics are summarized in **Supplementary Table S1**.

### Single Nucleotide Polymorphism Typing

**FIGURE 1:**
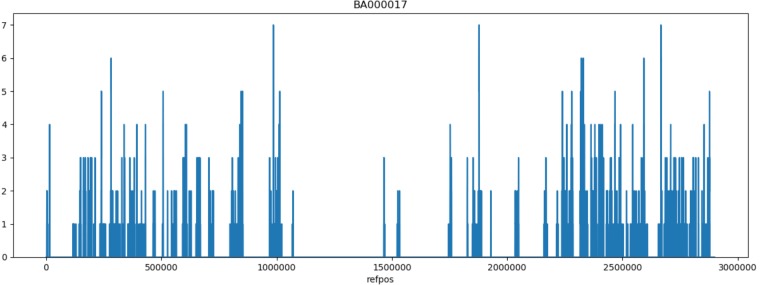
Genomic distribution of SNPs. The position of the 761 identified SNPs were plotted on the Mu50 chromosome using a sliding window of 1,000 bp. SNPs were distributed throughout regions included in the analysis and did not indicate any locations for mutational hotspots. Regions lacking SNP predictions are associated with locations of mobile genetic elements and repeat regions that were excluded from the SNP discovery and encode elements such as the SCC*mec* element and the β-hemolysin converting bacteriophage.

For reference based SNP discovery, genomes were aligned to the closed genome of reference strain Mu50, a vancomycin resistant HA-MRSA isolate from Japan (Hiramatsu et al., 1997). The core genome was determined by excluding identified MGEs and repeats. Core genome SNP discovery identified 764 SNPs comprised of: 154 intergenic, 186 synonymous, and 424 non-synonymous SNPs (**Supplementary Tables S2**, **S3**). Further evaluation of non-synonymous SNPs indicated that eight were shared by swine associated LA-MRSA ST5 isolates and not present in isolates from humans with no swine contact, as listed in **Table 1**. There were also two SNPs found only in MRSA ST5 isolates from humans with no swine contact (**Table 1**) that were specific to clinical isolates. The genes harboring the non-synonymous SNPs distinct to each subset of isolates have not been implicated in virulence of *S. aureus* and are unlikely to contribute to the pathogenicity of these isolates. SNPs were distributed throughout the Mu50 reference genome and no mutational hotspots were observed (**Figure 1**).

**Table 1:** Non-synonymous SNPs unique to LA-MRSA ST5 isolates or MRSA ST5 isolates from humans with no swine contact.

Number of swine associated isolates	Number of isolates from humans with no swine contact	SNP position^a^	Gene product^b^
82/82	0/72	160799	2′-3′-Cyclic-nucleotide 2′-phosphodiesterase
82/82	0/72	292343	Sorbitol dehydrogenase homolog
82/82	0/72	806489	Putative transporter
82/82	0/72	848820	Putative P-loop-containing kinase
82/82	0/72	1012841	Similar to ATP-dependent nuclease subunit A
82/82	0/72	1928498	*O*-Succinylbenzoic acid-CoA ligase
82/82	0/72	2695325	Ferrous iron transport protein B homolog
82/82	0/72	2720180	Regulatory protein
0/82	69/72	192929	Hypothetical protein
0/82	69/72	2277937	Conserved hypothetical protein

*^a^SNP position indicates the location of the SNP in the reference genome of Mu50. ^b^Gene product represents the protein produced by the gene containing the SNP of interest.*

### Phylogenetic Analysis

**FIGURE 2:**
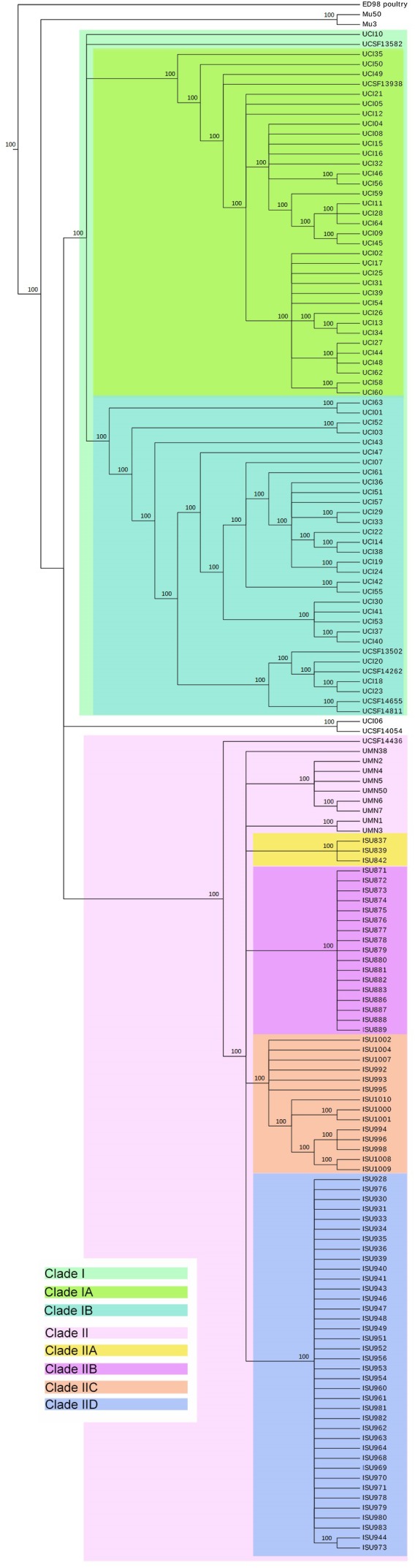
Maximum parsimony SNP tree of ST5 MRSA isolates. Comparison of 156 genomes, including 153 MRSA ST5 isolates, Mu3, ED98, and Mu50, yielded a total of 764 SNPs, of which 247 were parsimony informative. The tree shown is a majority-consensus tree of 4,440 equally parsimonious trees with a consistency index of 0.9428. Trees were recovered using a heuristic search in Paup 4.0b10 (Wilgenbusch and Swofford, 2003). This tree is broken into clades, with Clade I representing clinical MRSA ST5 isolates from humans with no swine contact and Clade II representing LA-MRSA ST5 isolates. Clade IIa–d are subsets of LA-MRSA ST5 isolates and each subclade represents an individual farm or production system.

**FIGURE 3:**
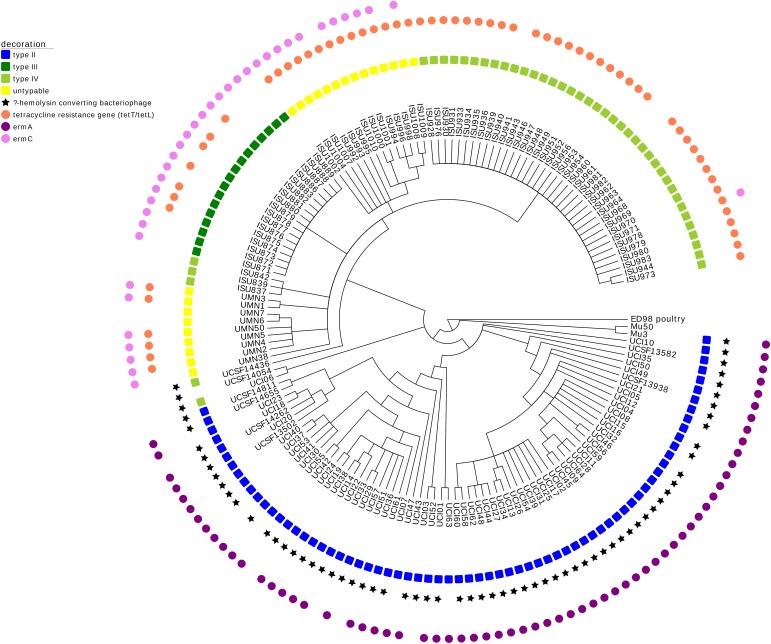
Maximum parsimony SNP tree of ST5 MRSA isolates with MGE analysis. The SNP tree developed for **Figure 2** was decorated using Evolview (He et al., 2016). The tree shows the MGE complement of these isolates, specifically describing the SCC*mec* element, the β-hemolysin converting bacteriophage (harboring virulence factors involved in innate immune evasion), and antimicrobial resistance genes involved in tetracycline resistance (*tetT/tetL*) and macrolide resistance (*ermA* and *ermC*).

A phylogenetic hypothesis was constructed from the identified core genome SNPs and rooted using the MRSA ST5 isolate Mu50. This tree depicts the evolutionary relationships between the 153 MRSA ST5 isolates and the reference isolates Mu3 and ED98 (**Figure 2**). The tree topology shows that swine associated LA-MRSA ST5 isolates cluster together and are separated from MRSA ST5 isolates from humans with no swine contact. These groups will be referred to as Clades I and II, representing MRSA ST5 isolates from humans with no swine contact and LA-MRSA ST5, respectively. A single MRSA ST5 isolate (UCSF14436) from a human with no swine contact was contained within Clade II. This isolate was the most distantly related of the Clade II isolates, harboring 24 unique/strain-specific SNPs. USCF14436 harbors a type IV SCC*mec* element and two AMR genes (*mecA* and *blaZ*), which is a strong indication this isolate is a CA-MRSA strain (**Figure 3**).

Methicillin resistant *S. aureus* ST5 isolates from humans with no swine contact (Clade I) are divided into subclades Ia and Ib. The SNP-level plasticity between MRSA ST5 isolates from humans with no swine contact is greater than that from LA-MRSA ST5 isolates. Despite the northern and southern geographic regions represented by the two groups of California isolates, the tree topology shows that UCSF isolates were found interspersed throughout Clade I and did not cluster by geographic location.

The swine associated isolates within Clade II formed four subclades, IIa–d. Each subclade represents a subset of isolates from a specific farm or farms; such that, Clade IIa represents isolates from Farm 10, Clade IIb represents isolates from Farm 24, Clade IIc represents isolates from Farm 46, and Clade IId represents isolates from Farms 38–42. Isolates originating from individual farms were genetically homogenous, possessing fewer than five SNPs (distinguishing them from other isolates from the same farm). These results suggest that LA-MRSA ST5 populations residing on farms are clonal and intermingling/transfer of isolates among farms or reintroduction of MRSA ST5 onto farms had likely not occurred. This was true for all swine associated isolates except those from Farms 38–42 (Clade IId). The high degree of genetic relatedness of the isolates within Clade IId suggests these farms are likely from a single production system or share a common genetic source, with exposure of pigs to LA-MRSA ST5 early in the production system (farrowing unit) and disseminated throughout the later stages as pigs are moved (finishing barns). High resolution profiling provides important phylogenetic signals for strain attribution (Eppinger et al., 2014; Rusconi et al., 2016). In this case, isolates from humans with short-term contact with swine farms could be traced back to a specific farm or production system. For example, isolates ISU886-ISU889 were traced to Farm 24, through SNP analysis. These isolates possessed the unique pattern of SNPs present on that farm or within that production system (Clade IIb, IIc, and IId), indicating exposure to the farm harboring that specific clone. The remaining isolates in Clade II include the isolates from humans with long-term swine contact and UCSF14436. Isolates from humans with long term swine contact did not cluster with isolates from the tested farms (**Figure 2**). This is consistent with the source of these isolates as it was unlikely the swine veterinarians sampled had contact with the specific farms sourcing the other swine associated isolates in this study. Though these isolates were distinct from the isolates obtained from swine, swine facilities, and humans with short term swine contact, they clustered together within Clade II and were representative of the livestock associated ST5 genotype. A Maximum-likelihood SNP tree was additionally constructed and revealed identical phylogenetic relationship between the MRSA ST5 isolates from humans with no swine contact and the swine-associated LA-MRSA ST5 isolates (**Supplementary Figure S1**).

### Mobile Genetic Element Analysis

The draft genomes of both LA-MRSA ST5 isolates and clinical MRSA ST5 isolates from humans with no swine contact were evaluated for MGEs. LA-MRSA ST5 isolates could also be distinguished from clinical MRSA ST5 isolates by the AMR genes and virulence factors they harbored (**Figure 3**). For LA-MRSA ST5 isolates, the SCC*mec* elements were of type III, IV, or untypable. Alternatively, clinical MRSA ST5 isolates harbored predominantly type II SCC*mec* elements and two type IV elements. Evaluation of the AMR genes revealed the primary macrolide resistance gene was *ermA* in clinical MRSA ST5 isolates, while LA-MRSA ST5 isolates harbored *ermC*. Additionally, tetracycline resistance genes were found exclusively in LA-MRSA ST5 isolates. Virulence factors harbored by the subsets were also different, with the majority (65/72, 90.3%) of clinical MRSA ST5 isolates harboring innate immune evasion genes within the β-hemolysin converting bacteriophage and none of the LA-MRSA ST5 isolates harboring these genes. This analysis revealed that MGE were not commonly shared between the two subsets of isolates.

## Discussion

Methicillin resistant *S. aureus* in livestock species was first identified in the 1970s (Devriese et al., 1972); however, discovery of a high prevalence of MRSA ST398 in swine in 2005, and subsequently in other livestock, brought debate over its public health implications to the forefront (Voss et al., 2005). Previous studies investigating LA-MRSA ST398 demonstrated these isolates have adapted to livestock hosts. This was represented by the absence of human specific virulence factors, such as the β-hemolysin converting bacteriophage (Price et al., 2012), and reduced adherence and transmission among humans (Graveland et al., 2011; Uhlemann et al., 2012). LA-MRSA ST9 isolates, similar to ST398, are thought to be livestock adapted, and clinical cases appear to be rare and attributed to animal contact (Cuny et al., 2015). While the ST5 lineage in humans is globally distributed and successful in both the hospital and community settings (Monecke et al., 2011), there are currently no reports of MRSA ST5 related disease occurring due to contact with livestock species; however, the genetic potential for swine isolates to adhere, invade, and cause disease in humans has not been investigated.

Here, we examined the phylogenetic relatedness applying high resolution core genome SNP profiling strategies on MRSA ST5 isolates from swine associated sources and humans with no swine contact to determine if distinct subpopulations of ST5 isolates exist within different host populations, which may provide evidence of host adaption. Similar analyses have been conducted for the ST398 lineage, where researchers identified that the studied LA-MRSA ST398 isolates were within the same clade and that clade appeared to have evolved from an ancestral human methicillin susceptible *S. aureus* (MSSA) ST398 clade (Price et al., 2012).

In this study, isolates from humans with no swine contact clustered distinctly from swine associated LA-MRSA ST5 isolates. All swine associated LA-MRSA ST5 isolates were contained within Clade II, while all but one MRSA ST5 from humans with no swine contact belonged to Clade I. The MGE complement of this clinical MRSA ST5 isolate (UCSF14436) suggests that it is likely a CA-MRSA isolate. CA-MRSA isolates tend to be genetically distinct from HA-MRSA isolates (Stefani et al., 2012), which may explain why UCSF14436 is found within the LA-MRSA clade. The separation of LA-MRSA ST5 isolates from clinical MRSA ST5 isolates based on their core genome relatedness supports our previous work, which indicated that swine associated LA-MRSA ST5 isolates harbor different MGEs containing virulence and resistance genes than MRSA ST5 from humans with no swine contact (Hau et al., 2015, 2017g; Hau, 2017). The data reported here further demonstrate the phylogenetic distinction of isolates into different groups and may reflect the adaptation of LA-MRSA ST5 isolates to colonization of swine. This distinction is based on focusing on MGEs harboring virulence factors and/or AMR and not on the full complement of all MGEs. This focus was warranted to begin evaluating the potential of LA-MRSA ST5 isolates to serve as a source for human infections or as a source for MGEs harboring virulence factors or AMR based on the isolates included in this study. Furthermore, similar to LA-MRSA ST398, evaluation of known virulence genes provides evidence that swine associated LA-MRSA ST5 may be less capable of causing disease in humans (**Figure 3**) (Hau et al., 2015). The *dN*/*dS* ratio observed within the core genome of the isolates was 2.3, which could potentially indicate that these strains recently evolved or underwent initial stages of diversification (Castillo-Ramirez et al., 2011).

Our results indicate frequent introduction of MRSA ST5 onto swine farms is unlikely and dominant clones of MRSA ST5 circulated within each farm at the time of sampling. A high degree of genetic relatedness was identified among swine-associated isolates. As expected, the population of isolates found on individual farms were dominated by clonal populations. This indicates either a single introduction of LA-MRSA ST5 onto a farm or dominance of a single variant that precludes colonization with alternative isolates introduced by human caretakers. However, multiple *S. aureus* lineages and spa types often occur simultaneously on swine farms and in individual pigs (Linhares et al., 2015), so the diversity of *S. aureus* in these animals may be underrepresented when looking specifically at MRSA ST5 isolates. This clonality was not present in isolates from humans with no swine contact, where genome plasticity was greater and individual isolates showed more diversity than that observed among LA-MRSA ST5 isolates.

While the evidence here suggests the populations of LA-MRSA ST5 and MRSA ST5 from humans with no swine contact are distinct, the isolates used in this analysis were sourced from geographically separated regions to ensure the clinical isolates were from patients with no swine contact. This design was utilized to build a solid reference point for comparing MRSA ST5 isolates from clinical and agricultural sources; however, because of the unlikelihood of these populations mingling, the SNPs identified can be attributed to either the geographic separation or the host species of the isolates. Future studies are warranted that include clinical MRSA ST5 isolates obtained from the hospital and community setting in areas of dense swine production, enabling further elucidation of the potential of LA-MRSA ST5 isolates to serve as a source for human infections or as a source for MGEs harboring virulence factors or AMR. As evidenced in this study, WGS followed by high resolution SNP profiling is a powerful molecular genomic epidemiology approach to gain insights into the population structure of LA-, CA-, and HA-MRSA and can provide important phylogenetic indicators for strain attribution.

## Author Contributions

SH, AA-G, BR, JH, PD, TF, ME, and TN conceived and designed the experiments, contributed reagents, materials, and analysis tools, and wrote the paper. SH, AA-G, and BR performed the experiments. SH, AA-G, BR, ME, and TN analyzed the data. All authors gave approval of the final version to be published and agreed to be accountable for all aspects of the work.

## Conflict of Interest Statement

The authors acknowledge receiving funding from the National Pork Board, however, declare that the funding sources in no way impacted experimental design, data analysis, manuscript preparation, or the decision to publish.

## Footnotes

1 http://mira-assembler.sourceforge.net/docs/DefinitiveGuideToMIRA.html

2 https://www.ncbi.nlm.nih.gov/genome/annotation_prok/

## Supplementary Material

FIGURE S1 Maximum-likelihood (ML) SNP tree of ST5 MRSA isolates obtained by RAxML. A Maximum-likelihood SNP tree was constructed from the 764 SNPs identified from the comparison of 156 genomes, including 153 MRSA ST5 isolates, Mu3, ED98, and Mu50.

TABLE S1 Genome sequencing statistics and MGE composition for all isolates used in study.

TABLE S2 General features linked to identified SNPs.

TABLE S3 Specific genomic features linked to identified SNPs.
