# Supplementary figures and images for: Single Nucleotide Polymorphism Analysis Indicates Genetic Distinction and Reduced Diversity of Swine-Associated Methicillin Resistant Staphylococcus aureus (MRSA) ST5 Isolates Compared to Clinical MRSA ST5 Isolates
Source: Front Microbiol. 2018 Sep 11;9:2078. doi: 10.3389/fmicb.2018.02078 (PMC6142820; doi:10.3389/fmicb.2018.02078)

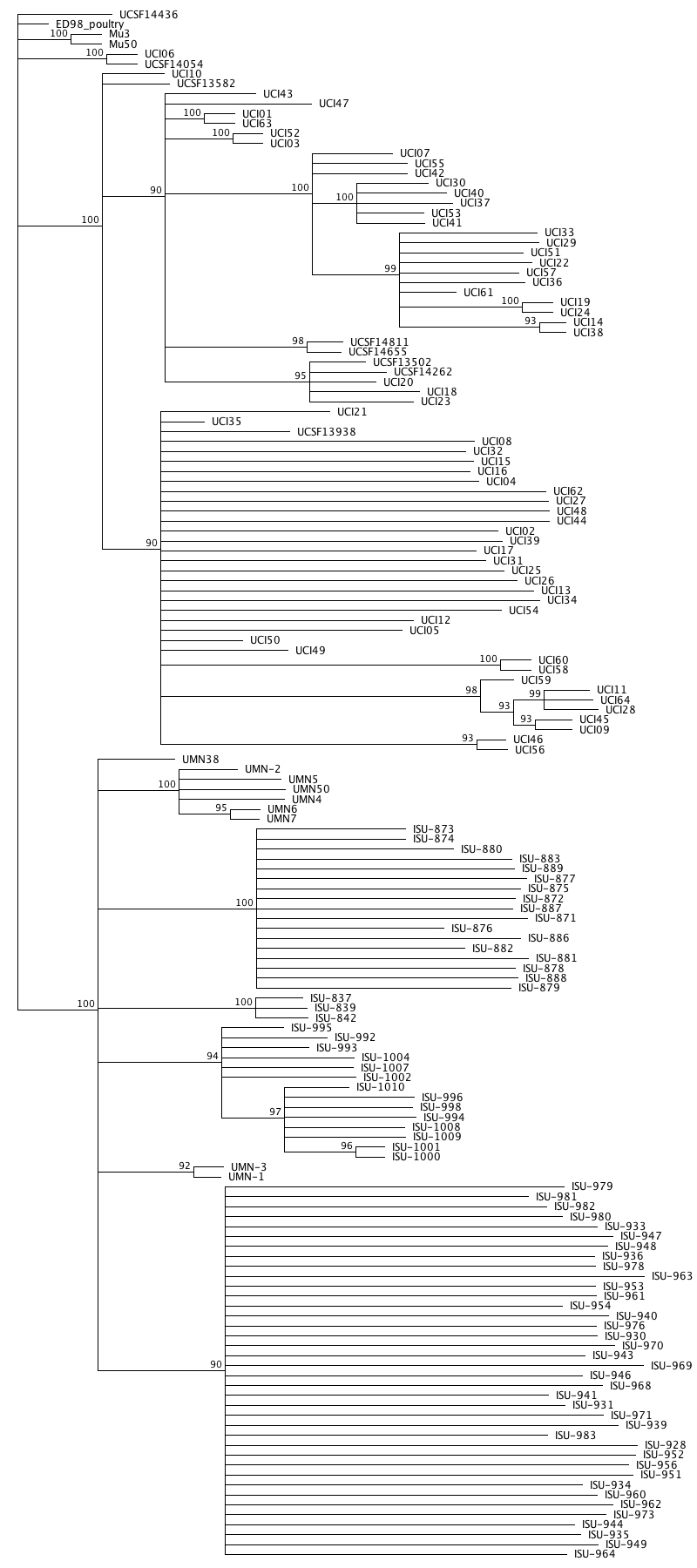

Supplement: FIGURE S1 — Maximum-likelihood (ML) SNP tree of ST5 MRSA isolates obtained by RAxML. A Maximum-likelihood SNP tree was constructed from the 764 SNPs identified from the comparison of 156 genomes, including 153 MRSA ST5 isolates, Mu3, ED98, and Mu50. [file Image_1.jpeg]
